# Covalent Organic Framework-Anchored Carbon Nanotubes Enabling Ultra-Thin Robust Polyimide Films for High-Specific-Power Flexible GaAs Solar Cells

**DOI:** 10.1007/s40820-026-02326-1

**Published:** 2026-08-10

**Authors:** Bin Li, Zhejun Guo, Qin Wang, Maoshu Yin, Xianglei Shi, Lu Yao, Chongjing Lu, Yanpeng Zhang, Wenwei Xu, Liying Jiang, Lijie Sun, Feng Shao, Xuan Liu, Nantao Hu

**Affiliations:** 1 Henan Key Laboratory of Information Functional Materials and Sensing Technology, Zhengzhou University of Light Industry, Zhengzhou, 450002 People’s Republic of China; 2 Research Center for Photovoltaics, Shanghai Institute of Space Power-Sources, Shanghai, 200245 People’s Republic of China; 3 College of Engineering Science and Technology, Shanghai Ocean University, Shanghai, 201306 People’s Republic of China; 4 Key Laboratory of Intelligent Creation for Extreme Energy Materials (Ministry of Education), Shanghai Jiao Tong University, Shanghai, 200240 People’s Republic of China; 5 Faculty of Chemical Engineering and Energy Technology, Shanghai Institute of Technology, Shanghai, 201418 People’s Republic of China

**Keywords:** Covalent organic frameworks, Carbon nanotubes, Polyimide, Composite films, High-specific-power flexible solar cell

## Abstract

Free-standing polymer films that are ultra-thin, lightweight, and robust hold significant promise for applications in flexible electronics. However, their performance has been constrained by the challenge of simultaneously enhancing mechanical strength and reducing density. Herein, an ultra-thin and robust polyimide (PI)-based covalent organic framework-modified multi-walled carbon nanotube (MWCNT-COF) film is demonstrated for high-specific-power flexible GaAs solar cells. The film exhibits a high Young’s modulus of 1.53 GPa, a tensile strength exceeding 97.53 MPa, and a low density of 0.73 g cm^−3^, representing a 45.1% reduction in density. The incorporation of 3D interconnected layered and porous MWCNT-COF significantly enhances the film’s mechanical properties while reducing its density. The synergistic effects of robust MWCNTs and covalently bonded COF layers, combined with the thermally stable PI molecular structure, impart exceptional strength and lightness to the film. Computational studies further confirm that COF-grafted MWCNTs effectively modify the polyamide acid matrix, substantially improving the mechanical properties of the PI films. Moreover, flexible dual-junction and triple-junction GaAs solar cells were successfully fabricated using 6 µm-thick MWCNT-COF/PI composite films, delivering outstanding specific powers of 8998 and 7749 W kg^−1^, along with efficiencies of 26.6% and 31.5% (AM0), respectively. These results underscore the potential for high-performance applications in space and energy systems. This work offers valuable insights into the development of high-performance flexible GaAs solar cells based on ultra-thin and robust PI films for a wide range of applications.

**Supplementary Information:**

The online version contains supplementary material available at 10.1007/s40820-026-02326-1.

## Highlights

- A scalable solvothermal route is proposed for directly growing covalent organic framework (COF) shells on carboxylated multi-walled carbon nanotube (MWCNT), yielding MWCNT-COF hybrids with high thermal stability.
- Polyimide composite films demonstrate a tensile strength of 97.5 MPa, a modulus of 1.53 GPa, and a density of 0.73 g cm⁻³, resolving the strength–density dilemma.
- Flexible dual-junction and triple-junction GaAs solar cells achieve ultrahigh specific powers of 8998 and 7749 W kg⁻¹, respectively, with minimal performance degradation after 2000 bending cycles at a 10 mm radius.

**Supplementary Information:**

The online version contains supplementary material available at 10.1007/s40820-026-02326-1.

## Graphical abstract

**Figure:**
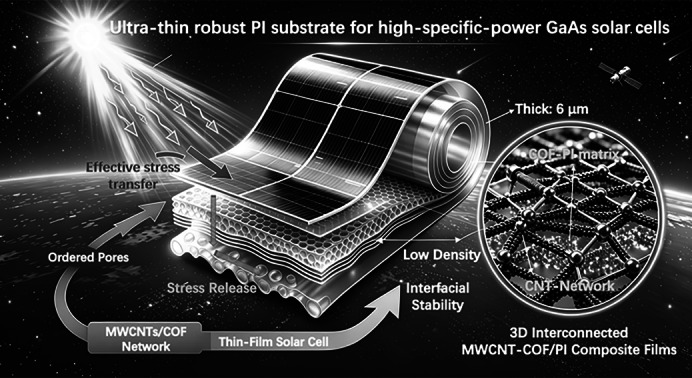

## Introduction

The rapid advancement of aerospace vehicles has created new demands for solar cell arrays that power these systems, particularly in terms of higher specific power and improved photoelectric conversion efficiency [1–3]. Flexible GaAs-based III–V solar cells are widely utilized in satellite and aerospace vehicle power systems due to their excellent spectral matching, high photoelectric conversion efficiency, and robust mechanical strength [4]. For aerospace applications, especially in constrained spaces, achieving high specific power is essential without compromising performance [5–7]. Consequently, ultra-thin flexible GaAs solar cells are emerging as a key development direction for the future. However, current flexible substrates typically have densities exceeding 1 g cm^−3^, limiting the potential to reduce the mass of flexible thin-film solar cells. Therefore, optimizing the density of the flexible substrate while ensuring its mechanical compatibility with the ultra-thin photoactive layer is critical for enhancing the specific power of flexible GaAs solar cells [8].

To fulfill the stringent requirements for flexible GaAs solar cell substrates, high-performance polymers such as polyimide (PI) are widely utilized due to their excellent mechanical properties and thermal stability [9, 10]. While conventional PI films exhibit high strength, they typically have densities exceeding 1.3 g cm^−3^, posing a significant challenge for further weight reduction. This issue is particularly critical in weight-sensitive applications, including space solar arrays, drone-integrated photovoltaics, and wearable electronics, where substrate mass directly impacts launch cost, endurance capability, and wearing comfort. In response to the growing demand in microelectronics, aerospace, and renewable energy, there is an urgent need to develop porous PI films with low densities that do not compromise mechanical strength [11–14]. However, introducing porosity often leads to significant deterioration in mechanical properties, and higher porosity inevitably results in greater mechanical loss. Thus, resolving the inherent trade-off between density reduction and mechanical reinforcement is a central challenge. The fabrication of ultra-thin and robust PI films is essential to meet the combined requirements of mechanical strength, thickness, and low-density for various high-performance applications.

To enhance the mechanical properties of porous PI films, hybrid films incorporating carbon nanotube (CNT) fillers are commonly employed [15, 16]. For instance, Lu et al. developed a series of films that demonstrated flexibility, a low coefficient of thermal expansion (CTE), and improved mechanical properties through the introduction of carboxylated carbon nanotube (C-CNT) [17]. The C-CNT/PI composite films exhibited the lowest CTE and the highest mechanical properties among the tested samples, along with enhanced thermal stability. Despite these advances, the mechanical performance of CNT-enhanced porous PI film remains insufficient. This limitation can be attributed to three primary factors: (i) severe agglomeration of CNTs within the PI matrix, particularly constrained by the porous structures, which leads to stress concentration and defects; (ii) weak interfacial interactions between pristine or carboxylated CNTs and the PI matrix, hindering efficient load transfer from the polymer to the reinforcing filler; and (iii) the challenge of constructing a continuous three-dimensional (3D) reinforcement network using conventional physical blending methods, which significantly restricts the reinforcement efficiency of CNTs. Therefore, novel strategies are urgently required to achieve uniform dispersion of CNTs and efficient load transfer within the PI matrix through rational interfacial design and architectural engineering.

Covalent organic frameworks (COFs), a class of porous crystalline materials composed of light elements (such as C, N, O) linked by strong covalent bonds, have recently garnered attention due to their unique properties, which include uniform pore sizes, customizable surface functional groups, ordered channel structure, and excellent thermal and chemical stability [18, 19]. Incorporating COFs into polymer films is anticipated to impart low-density and porous characteristics while preserving or even enhancing mechanical properties. Zhao et al. introduced COFs into PI films, resulting in cross-linking networks that enhanced the breakdown strength and thermal stability of the PI matrix [20]. Similarly, Li et al. demonstrated that COF nanospheres embedded in polymer films improved their mechanical flexibility and self-supporting capability [21]. However, despite these promising advancements, two critical issues remain unresolved: (i) most COF materials exhibit lower thermal stability than PI, which can compromise the high-temperature performance of COF/PI composites; and (ii) existing studies have primarily focused on bare COFs or simple COF/polymer blends, without fully exploiting the synergistic reinforcement effects between CNTs and COFs. Additionally, by combining CNTs with COFs through a coaxial core–shell structure, a synergistic effect can be achieved. In this configuration, the CNTs serve as the polymer framework, providing mechanical support, while the COFs can be chemically bonded to the surface of the CNTs to create covalent interfacial bonds. This ensures effective stress transfer and ultimately results in a more robust composite film.

To address these research gaps, we present a strategy that leverages covalently bonded coaxial MWCNT-COF hybrids incorporated into a PI matrix via in situ polymerization, successfully fabricating ultra-thin, robust, and ultra-lightweight MWCNT-COF/PI composite films for high-specific-power flexible GaAs solar cells. The ultra-thin MWCNT-COF/PI composite films were facilely formed by introducing the obtained porous MWCNT-COF coaxial hybrids into a poly (amic acid) (PAA) precursor solution through in situ polymerization processes. The resulting ultra-thin porous MWCNT-COF/PI composite films exhibited significant enhancements in mechanical properties, including a 109% increase in Young’s modulus and a 35.4% increase in tensile strength compared to bare PI films. Moreover, the density of the composite films was reduced by 45.1%. It’s suggested that these improvements are attributed to the 3D interconnected layered and porous nature of the MWCNTs-COF composites, which significantly contribute to the high specific power of flexible GaAs solar cells. High efficiencies of 26.6% and 31.5% (AM0) were achieved for the flexible dual-junction and triple-junction GaAs solar cells based on 6-µm-thick MWCNT-COF/PI composite film substrates, enabling ultra-high-power densities of 8998 and 7749 W kg^−1^, respectively. This work presents a promising approach for developing high-specific-power flexible GaAs solar cells with ultra-thin and ultra-strong properties, thereby opening new possibilities for their application in various fields.

## Experimental Section

### Materials

MWCNTs (95%) were obtained from Glatt (Shanghai) Information Technology Co., Ltd. 3, 3',4,4'-Biphenyl tetracarboxylic acid dianhydride (s-BPDA, 97%) and 4,4'-diaminodiphenyl ether (ODA, 99.45%) were purchased from Shanghai Bide Pharmaceutical Technology Co., Ltd. N-methylpyrrolidone (NMP, 99.5%) and Pyromellitic dianhydride (PMDA, 96%) were sourced from Shanghai Maclean's Biochemical Technology Co., Ltd. N, N-dimethylacetamide (DMAc, 99.5%) and Methyl acrylate (MA, 99%) were purchased from Shanghai Aladdin Biochemical Technology Co., Ltd. Concentrated sulfuric acid (H_2_SO_4_, 95%) and concentrated nitric acid (HNO_3_, 65%) were purchased from Sinopharm Chemical Reagent Co., Ltd. Aminopropyl double-terminated dimethicone (APPS, 99%) and absolute ethanol (C_2_H_5_OH, 99.7%) were purchased from Shanghai Merrell Biochemical Technology Co., Ltd.

### Purification and Carboxylation of MWCNTs

The MWCNT powders were initially oxidized in air and subsequently refluxed in a concentrated sulfuric acid/nitric acid mixture (3:1 v/v) to remove metal catalyst impurities and introduce carboxyl groups onto the MWCNT surface. Typically, 600 mg of MWCNTs were heated in an oven at 300 °C for 1 h. The thermally treated MWCNTs were then transferred to a 100 mL three-neck flask equipped with a reflux condenser, to which 30 mL of concentrated sulfuric acid and 10 mL of concentrated nitric acid were added. The mixture was heated and magnetically stirred at 60 °C for 12 h. After cooling to room temperature, the suspension was diluted with 300 mL of deionized water and allowed to stand until the MWCNTs had completely precipitated. The supernatant was decanted, and the residue was further diluted with another 300 mL of deionized water, filtered, and washed until the filtrate was neutral. The resulting black MWCNT filter cake was then vacuum-dried at 60 °C for 24 h to yield carboxylated MWCNTs (MWCNT-COOH).

### Synthesis of PI-Based COF and COF-Modified MWCNTs

During the polymerization process of siloxane block polyimide (API), 1 to 5 wt% APPS were added. After thermal imidization to form the film, API-1 to API-5 films were obtained, which were named API-1, API-2, API-3, API-4, and API-5, respectively. In addition, a total of 136 mg of MWCNT-COOH powders were dispersed in 100 mL of NMP via sonication for 1 h. Subsequently, 983 mg of PMDA and 165 μL of deionized water were added, and the mixture was heated and stirred at 80 °C for 2 h. After cooling to room temperature, the suspension was subjected to further sonication. Then, 378 mg of MA dissolved in 20 mL of NMP was added dropwise under sonication, and the mixture was stirred at room temperature for 6 h. The resulting solution was transferred to an oven and maintained at 210 °C for 24 h. After cooling to room temperature, the solid products were collected by suction filtration and sequentially washed with NMP, absolute ethanol, and deionized water. The final MWCNT-COF solids were freeze-dried for 36 h. For comparison, the bare PI-based COF (PI-COF) powders were synthesized using the same procedure without the addition of MWCNT-COOH [22].

### Preparation of Composite Films

Firstly, 1 mg of MWCNT-COF powder was dispersed in 10 mL of DMAc using ultrasonication for 1 h. Then, 396 mg of ODA and 40 mg of APPS were added to form a mixed solution. Subsequently, 596 mg of s-BPDA was added in batches. The reaction solution was stirred at 30 °C for 5 h to obtain the MWCNT-COF/PAA composite precursor solution. The precursor solution was cast onto a glass plate using a 250 µm scraper to form a film. The film was then heated programmatically at 60, 90, and 120 °C, each for 30 min. Following this, the film underwent imidization at 150, 180, and 300 °C, each for 1 h. After peeling the film off the glass substrate, the MWCNT-COF/PI film was obtained (denoted as 0.1 wt% MWCNT-COF). Depending on the varying masses of MWCNT-COF added to the solution, the samples with MWCNT-COF amounts of 1, 2, 5, 10, and 20 mg were named 0.1 wt% MWCNT-COF, 0.2 wt% MWCNT-COF, 0.5 wt% MWCNT-COF, 1 wt% MWCNT-COF and 2 wt% MWCNT-COF, respectively.

### Theoretical Calculations

The computational approach was as follows: molecular structures were constructed in GaussView 6 and their geometries were optimized within Gaussian 16 using density functional theory (DFT) at the B3LYP/6-31G level. For more accurate energy calculations, single-point energy calculations were performed on the optimized structures using the larger 6–311 + G (d, p) basis set. Throughout this process, the scf = xqc keyword was employed to ensure the convergence of the self-consistent field procedure.

### Characterization Methods

Powder samples were analyzed using Fourier transform infrared spectroscopy (FTIR, Thermo Scientific Nicolet iS20, USA) in ATR mode for film samples. The UV–Vis spectra were obtained with a UV–VIS spectrophotometer (UV-3600i Plus, Japan). For surface analysis of all powder samples, Raman spectroscopy (Horiba Lab RAM HR Evolution, Japan) with a 532 nm laser wavelength, as well as X-ray photoelectron spectroscopy (XPS, Thermo Scientific K-Alpha, USA) were employed. The morphology and elemental composition were examined using a scanning electron microscope (SEM, ZEISS Gemini SEM 360, Germany, 5 kV), while the microstructure of the materials was characterized by atomic force microscopy (AFM, FastScan Bio, USA) operating in tapping mode, and transmission electron microscope (TEM, JEOL JEM-F200, Japan) at a voltage of 200 kV. Thermal stability was evaluated using thermogravimetric analysis (TGA, Netzsch STA 449 F5, Germany), with the temperature ramped from 25 to 800 °C at a rate of 10 °C min^−1^ in a nitrogen atmosphere. Mechanical properties were assessed with an electronic universal testing machine (INSTRON3367, USA), using film samples with dimensions of 15 mm × 10 mm, each tested five times. The static water contact angle was measured using a video contact angle tester (Chengde Dingsheng JY-82C, China). The current–voltage (J-V) characteristics and external quantum efficiency (EQE) of solar cells were measured with standard solar cell testing equipment (Spectrolab X25A Mark II, China) under AM0 conditions at 25 °C. For density measurement of the films, a precision balance was used to obtain the film weight and a vernier caliper to measure the film thickness. The volume of the film was then calculated, and the density was obtained using the appropriate density calculation formula.

## Results and Discussion

### Design and Structure of MWCNT-COF/PI Composite Films

**Fig. 1:**
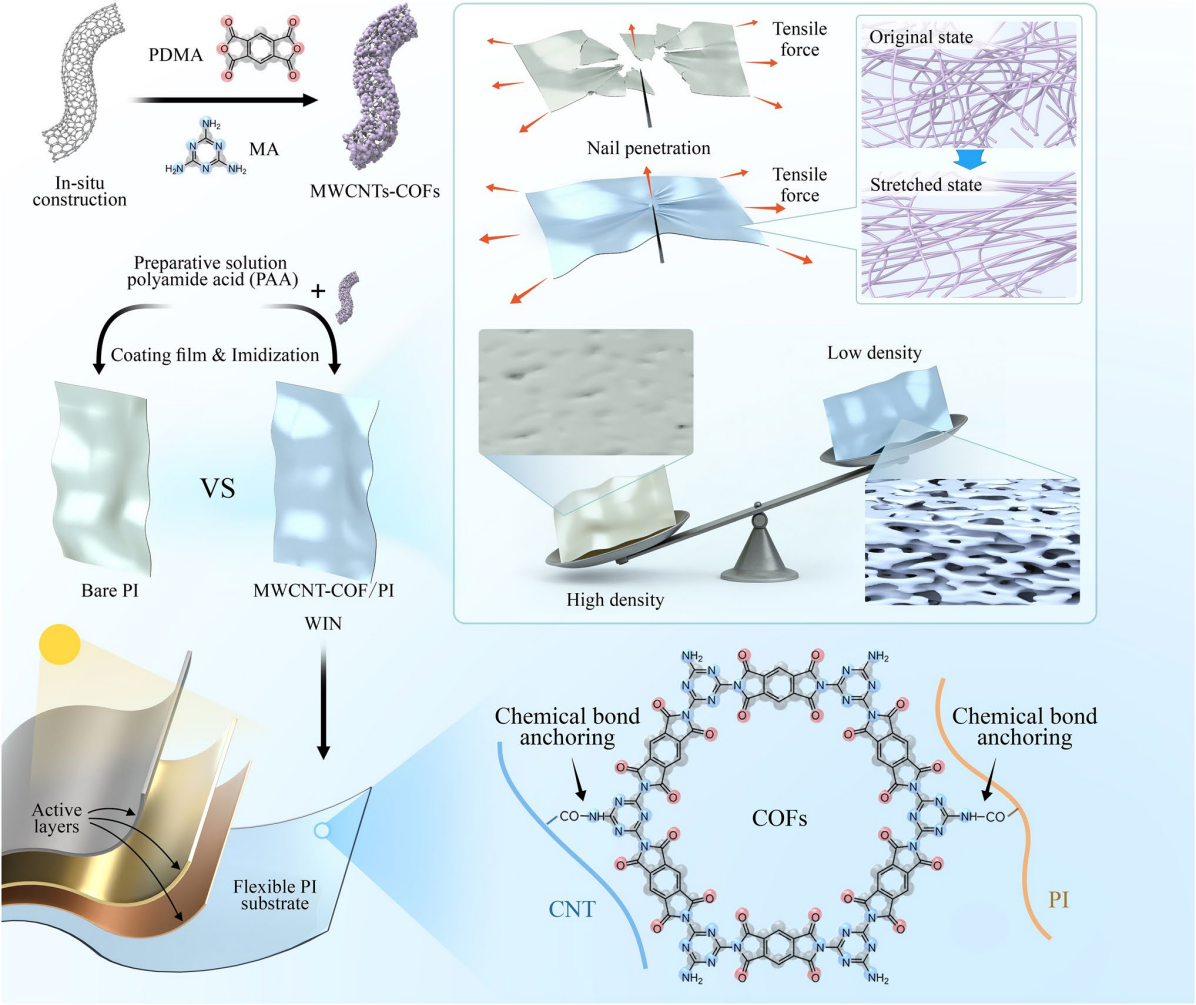
Schematic illustration of synthesis process and the ultra-thin, robust PI film structure formed through the anchoring of CNTs by COFs

The synthesis process for the MWCNT-COF coaxial hybrid material and MWCNT-COF/PI composite films is shown in Figs. 1 and S1. Initially, MWCNT-COOH was prepared to ensure the uniform distribution of COF materials on the MWCNT surface. During the solvothermal process, the polymerization of MA and PDMA occurred, resulting in the formation of a porous PI-based COF layer that is covalently attached to the MWCNT surface. The resulting MWCNT-COF coaxial hybrid was then introduced into the siloxane-containing PAA (s-BPDA/ODA/APPS) solution, forming a well-dispersed MWCNT-COF hybrid suspension within the PAA precursor. Following successive film casting, drying, and thermal imidization steps, ultra-thin, porous MWCNT-COF/PI composite films were obtained. Compared to traditional bare PI films, this ultra-thin and robust PI film structure can withstand lateral tensile forces and longitudinal nail penetration to a certain extent, thanks to the anchoring effect of chemical bonds from both CNTs and COFs within the PI substrate. Moreover, the interwoven layered distribution of the 3D CNT composite network imparts a degree of porosity to the PI substrate, which greatly reduces the density of the flexible PI substrate. This type of PI film, characterized by low density, light weight, and high toughness, is ideally suited as a base for flexible solar cells, offering high power-to-weight ratios and providing continuous energy for wearable devices and aerial vehicles.

**Fig. 2:**
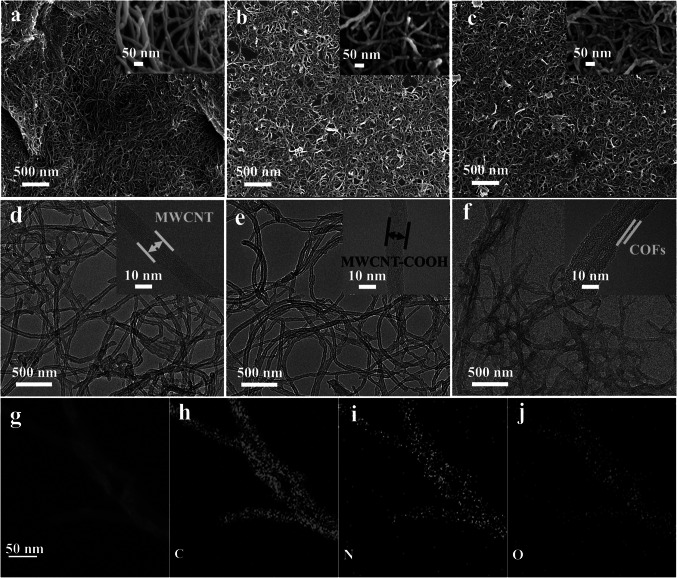
Characterization of composite materials. SEM images of **a** MWCNT, **b** MWCNT-COOH, and **c** MWCNT-COF hybrids, with insets showing magnified views of each; TEM images of **d** MWCNTs, **e** MWCNT-COOH, and **f** MWCNT-COF hybrids, with insets showing the surface morphology of MWCNTs with different surface modifications; EDS mapping images of MWCNT-COF:** g** SEM**, h** C**, i** N and** j** O

**Fig. 3:**
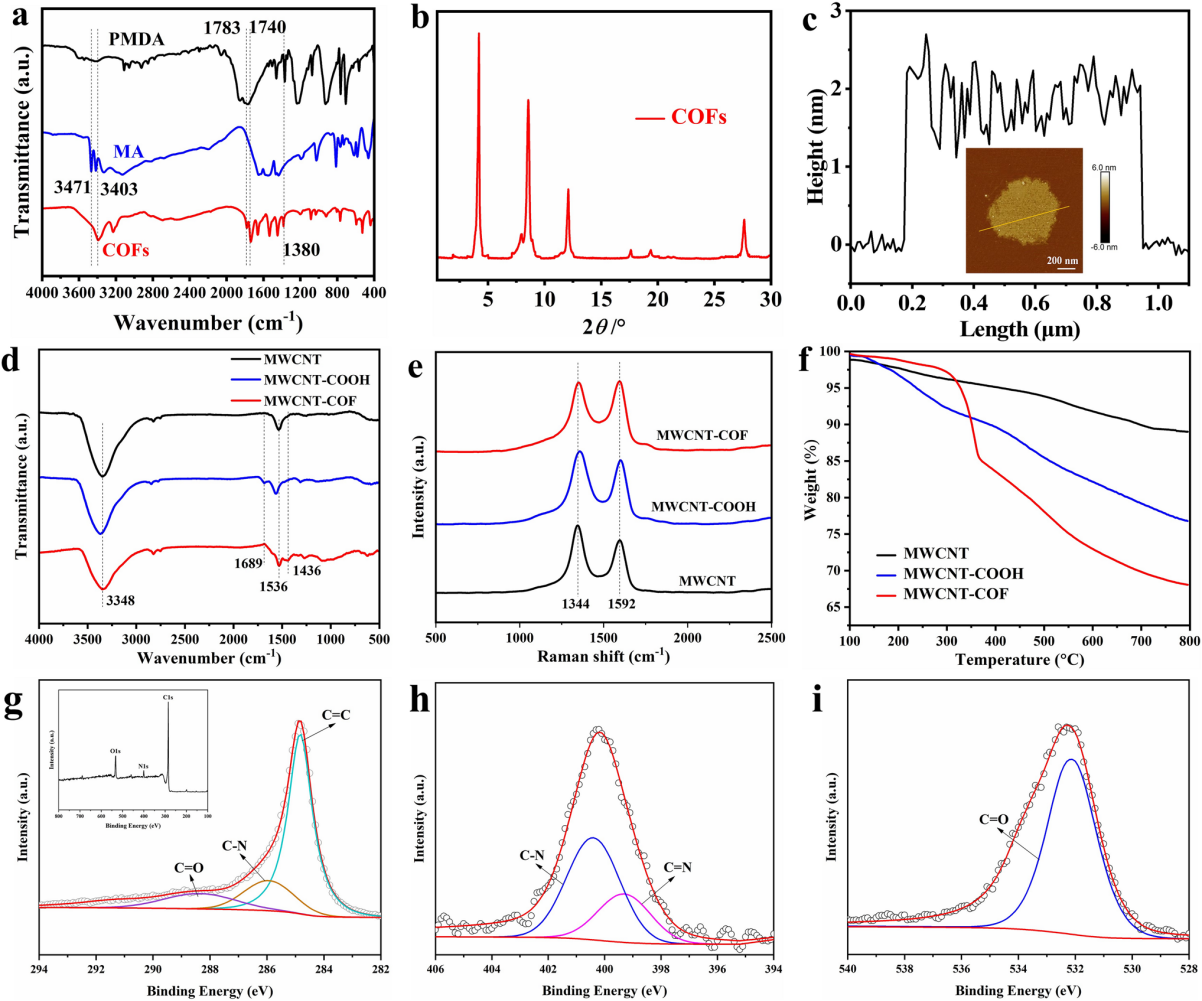
Structure of MWCNT-COF/PI composite films. **a** FTIR spectra of PDMA, MA, and COFs, **b** XRD patterns of COFs nanosheets, **c** AFM images of COF nanosheets (Height profile of COF nanosheets); **d** FTIR spectra, **e** Raman spectra and **f** TGA curves of MWCNT, MWCNT-COOH, and MWCNT-COF hybrids; XPS spectra of MWCNT-COF: **g** C 1*s*, **h** N 1*s*, **i** O 1*s*

To investigate the source of ultra-light and ultra-strong properties of MWCNT-COF/PI composite films, the microstructures of the composite films were examined through SEM characterization. The SEM images of MWCNT, MWCNT-COOH, and MWCNT-COF hybrids are displayed in Fig. 2a–c. As shown in Fig. 2a, MWCNT features a smooth surface, while the carboxylated MWCNT exhibits a rough surface, reduced length, and a scattered distribution. In contrast, the MWCNT-COF hybrid (Fig. 2c) exhibits a rougher surface, indicating the successful growth of COFs on the MWCNT surface [23]. As shown in Fig. S2a–f, the upper surface of the MWCNT-COF/PI composite films develops a uniformly dispersed network structure as the content of MWCNT-COF hybrid increases, which is conducive to obtaining ultra-light and ultra-strong properties of composite films. TEM images of MWCNTs, MWCNT-COOH, and MWCNT-COF hybrids are shown in Fig. 2d–f. It can be clearly observed that the lattice fringes of MWCNT-COOH display a smooth surface (Fig. 3e), whereas the MWCNT-COF surface (Fig. 3f) is characterized by the chemical bonds of PI-COFs, resulting in a rougher structure and a more robust composite film. EDS results (Fig. 3g–j) indicate a uniform distribution of C, N, and O elements throughout the MWCNT-COF hybrid, confirming the successful growth of PI-COFs on MWCNTs, which is consistent with the SEM observations.

The FTIR spectra and XRD spectra of the COF materials are shown in Fig. 3a, b. The diffraction peak of XRD spectra at 4.19° is attributed to the (100) plane, indicating that the COFs possess a crystalline structure with a 2D ordered layered arrangement. The peaks at 8.55° and 12.07° are attributed to the (110) and (210) planes, respectively. Furthermore, the peak at 27.66° correlates with the (001) plane, suggesting the existence of interlayer π-π stacking between the layers of COFs. The AFM images of the PI-COF nanosheet (Fig. 3c) reveal that the thickness of the 2D PI-COF nanosheet structure is approximately 2 nm. The FTIR spectra of MWCNT, MWCNT-COOH, and MWCNT-COF materials are shown in Fig. 3d. We can see that the peak at 3348 cm^−1^ corresponds to the vibrational mode of the hydroxyl groups on the MWCNTs. The characteristic peak at 1689 cm^−1^ is attributed to the carboxyl group, confirming the successful carboxylation of the MWCNTs. Additionally, the vibrational peak at 1536 cm^−1^ represents the E_1u_ vibrational mode of the MWCNT wall, indicating the presence of a graphite structure. In MWCNT-COF, the peak at 1436 cm^−1^ is assigned to the C–N bond in the imide formed by the condensation reaction of PMDA and MA, demonstrating the formation of a COF linked by C–N bonding [22]. The Raman spectra (Fig. 3e) indicate that the D band and G band of MWCNT-COF appear at 1344 and 1592 cm^−1^, respectively. The intensity ratio of the D band to the G band for MWCNT-COF decreases from 1.254 to 0.982 compared to that of MWCNT, confirming that the in situ grown COF layer successfully encapsulated the CNTs and may passivate surface defects through chemical bonding. Meanwhile, the G band exhibits a positive shift, indicating a strong interaction between PI-COFs and MWCNTs in the MWCNT-COF structure [22]. To meet the thermal cycling characteristics of the composite films in space, the TGA diagrams of MWCNT, MWCNT-COOH, and MWCNT-COF powder materials are displayed in Fig. 3f. It can be observed that the weight loss of MWCNT is only 10%, indicating excellent thermal stability. After acidification treatments, the weight loss increases to 22%, confirming the presence of 13.3 wt% carboxyl functional groups. However, upon further modification of the MWCNTs with COF layers, the weight loss increases to 32.5%, corresponding to approximately 19.2 wt% COF layers. Within the temperature range of 300 ~ 400 °C, there is a noticeable weight loss for MWCNT-COF, which is attributed to the significant thermal decomposition of the amino functional groups present in the MWCNT-COF. The valence states of each element in the MWCNT-COF hybrid were further analyzed by XPS. The C 1*s* spectrum (Fig. 3g) reveals peaks corresponding to C=C, C–N, and C=O bonds. The N 1*s* spectrum (Fig. 3h) shows two characteristic peaks at 399.3 and 400.4 eV, attributed to C–N bonds in the COF and C=N bonds in the MA unit, respectively. The O 1*s* spectrum (Fig. 3i) displays a single characteristic peak corresponding to C=O bonds on the MWCNT surfaces [24].

### Thermodynamic and Physical Properties of Composite Films

**Fig. 4:**
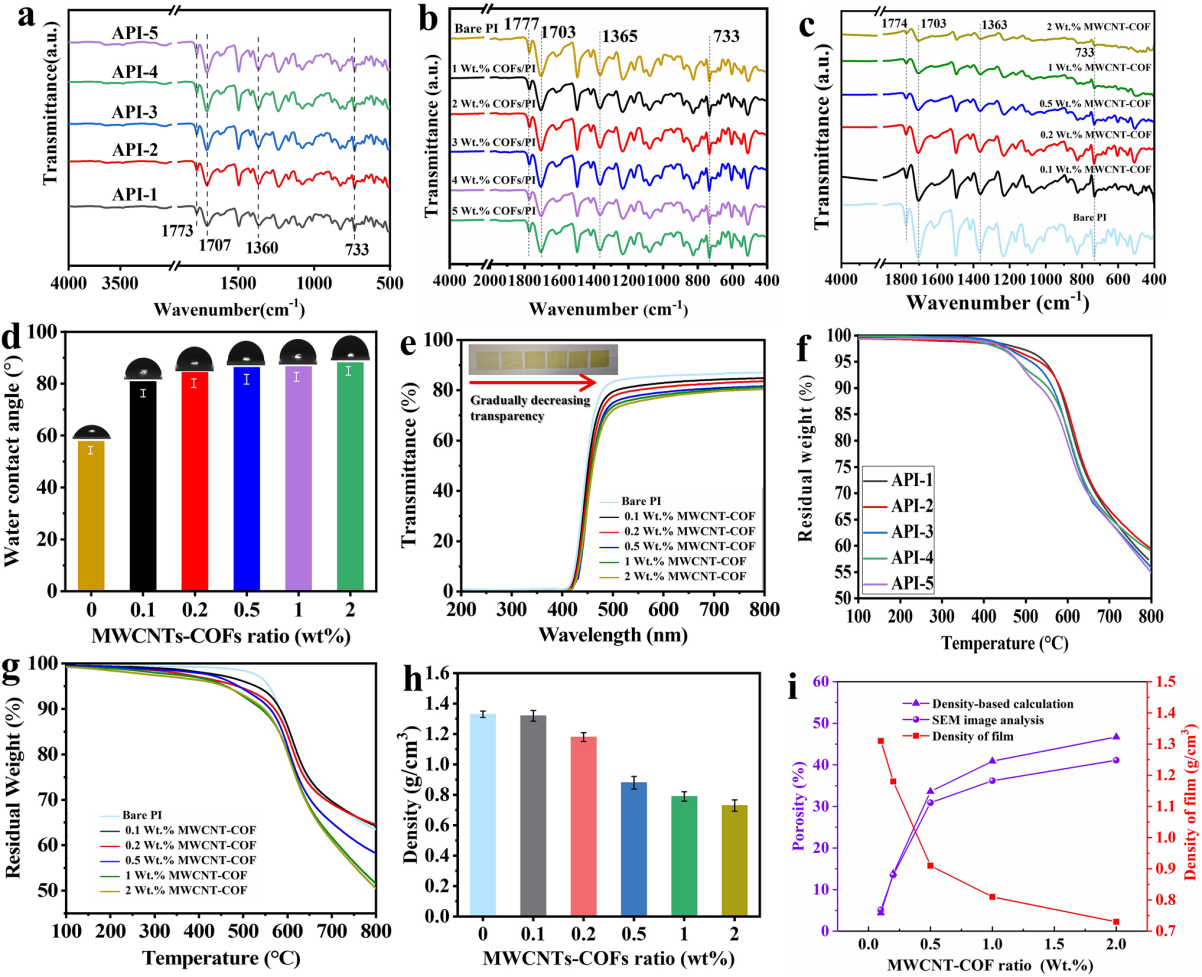
Surface and functional properties of bare PI and MWCNT-COF/PI composite films. FTIR spectra of **a** PI, **b** COFs/PI, and **c** MWCNT-COF/PI composite films;** d** Water contact angles of bare PI and MWCNT-COF/PI composite films; **e** UV–Vis spectra of bare PI and MWCNT-COF/PI films; TGA curves of **f** API films and **g** MWCNT-COF/PI composite films with varying contents; **h** Comparison of density values; **i** Comparison of porosity in film density using density-based calculations and SEM image analysis

Moreover, the FTIR spectra of API, COFs/PI, and MWCNT-COF/PI composite films (Fig. 4a–c) display characteristic peaks of the imide groups. The symmetric and asymmetric stretching vibrations of C=O at 1703 and 1774 cm^−1^, the stretching vibration of C–N at 1363 cm^−1^, and the bending vibration of C=O at 733 cm^−1^ confirm the successful imidization of PI. Notably, no characteristic peaks corresponding to the PAA intermediate (1660 cm^−1^) are observed, indicating complete conversion [25]. Additionally, the characteristic peaks between 1000 and 1100 cm^−1^ correspond to the Si–O–Si stretching vibrations, demonstrating the successful incorporation of APPS into the PI molecular chain [25]. In practical applications, such as solar cells, composite substrates inevitably encounter water vapor in their environment. The failure of these devices is often linked to water absorption and the subsequent cracking of the polymer substrate, ultimately leading to severe performance degradation. Therefore, enhancing the surface hydrophobicity of polyimide films is essential for improving their environmental stability. The water contact angle of bare PI films and MWCNT-COF/PI composite films was measured, with the results shown in Figs. S3 and 4d. The bare PI film exhibits a static water contact angle of 57.8°, indicating its hydrophilic nature. Upon the introduction of the MWCNT-COF hybrid, the contact angle gradually increases with rising filler content. When the MWCNT-COF loading reaches 2 wt%, the contact angle exceeds 80°, demonstrating that the incorporation of the MWCNT-COF hybrid significantly enhances the surface hydrophobicity of PI films. This improvement effectively reduces the condensation of atmospheric moisture on the substrate, thereby minimizing the adverse effects of water vapor on electronic devices. The optical transmittance of the composite substrates was also investigated in order to obtain better light conversion characteristics for solar cells. As shown in Fig. 4e, PI films with varying composite ratios exhibit high transmittance across the wavelength range of 500–800 nm.

The TGA analysis of the composite films (Fig. 4f, g) indicates that the addition of MWCNT-COF into the PI matrix does not significantly alter the degradation mechanism of the PI films. All films exhibit similar thermogravimetric curves, demonstrating good thermal stability, with no significant mass loss observed below 500 °C. In addition, the cross sectional SEM images of bare PI, COFs/PI, and MWCNT-COF/PI composite films are presented in Figs. S4a–f and S5a–f. As the content of MWCNT-COF increases, the 3D interconnected layered structure becomes more prominent, accompanied by a reduction in density due to the 3D network arrangement of the CNT composites. The CNTs anchored with COF are embedded within the polymer, forming a porous, layered structure. Moreover, the incorporation of the 3D interconnected layered and porous MWCNT-COF/PI composites not only enhances the tensile strength, flexibility, and other mechanical properties of the films but also significantly reduces their weight, thereby improving the power density of electronic devices such as solar cells.

The density values of composite films are shown in Fig. 4h. As the doping ratio of MWCNT-COF increases, the porosity of the films gradually rises, while their density gradually decreases. Notably, the density of the 2 wt% MWCNT-COF/PI composite films drops to 0.73 g cm^−3^ (compared to 1.33 g cm^−3^ for the bare PI films), representing a 45.1% decrease in density following the addition of MWCNT-COF hybrids. This emphasizes the crucial role of the 3D interconnected layered and porous MWCNT-COF coaxial hybrid in improving the performance of PI films. Moreover, we performed a quantitative porosity analysis using both density-based calculations and SEM image analysis. Figure 4i shows the calculated porosity for different doping ratios of MWCNT-COF, along with a comparison to the change in film density derived from both methods including density-based calculation and SEM image analysis (Fig. S6a-e). The increasing trend in porosity calculated by both methods is consistent. The slight differences in porosity values between the two methods can be attributed to the fact that the highly doped and low-density MWCNT-COF occupies more space within the original PI substrate, resulting in slightly lower values obtained through SEM image analysis compared to density-based calculations. The MWCNTs coated with COF are embedded within the polymer, forming a porous structure that significantly reduces the film’s weight while enhancing its mechanical properties. The quantitative results confirm that the observed density reduction (from 1.31 to 0.73 g cm^−3^) is attributable to the highly porous structure introduced by the MWCNT-COF network.

### Mechanisms of Mechanical Reinforcement in Composite Films

**Fig. 5:**
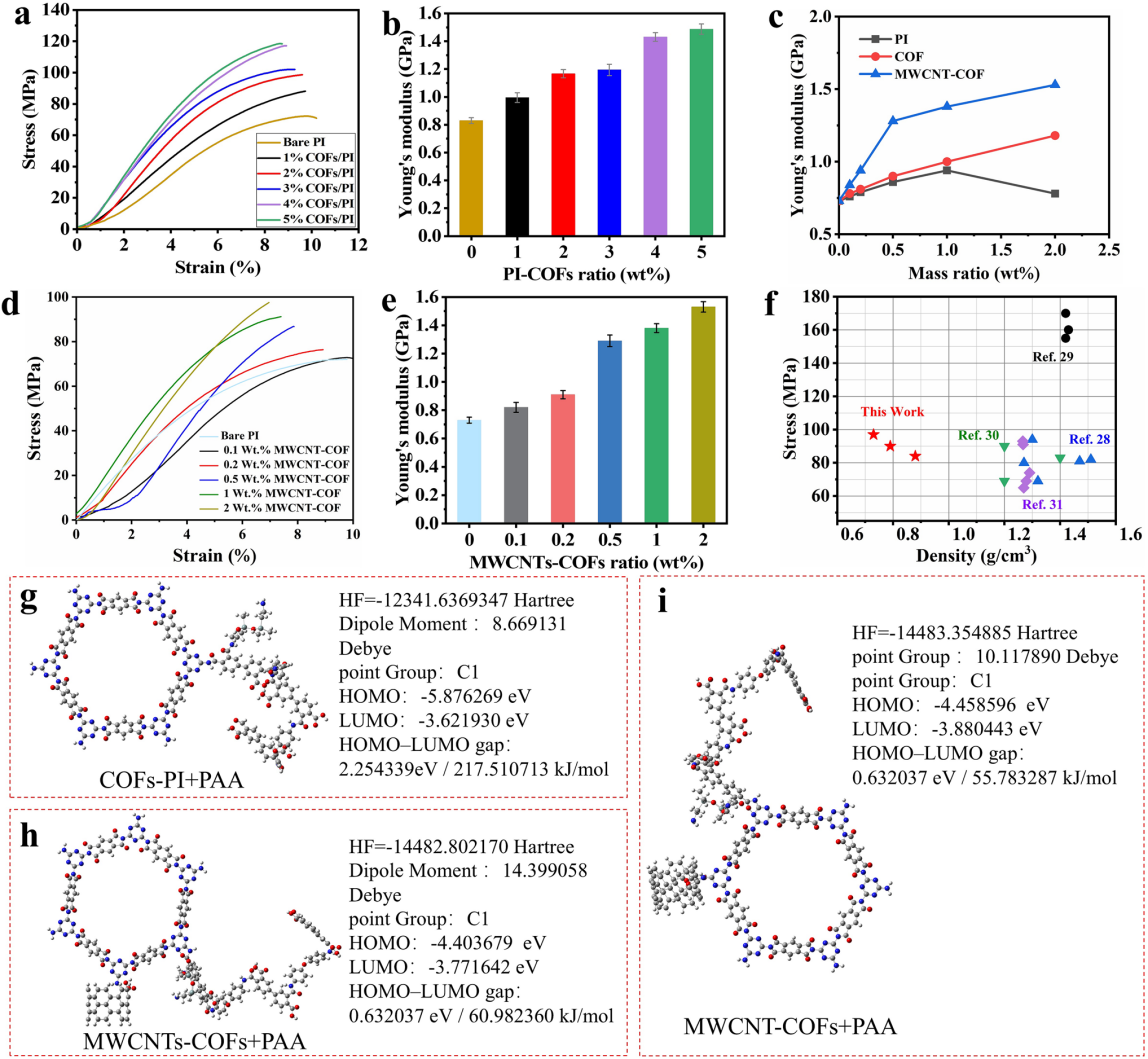
Properties of bare PI and MWCNT-COF/PI composite films with varying MWCNT-COF contents and Gaussian calculation results. **a** Stress–Strain curves and **b** Young's modulus of COFs/PI films; **c** Young's modulus comparison plots of PI, COF, MWCNT-COF/PI films; **d** Stress–strain curves, **e** Young's modulus plot of MWCNT-COF/PI composite films with varying MWCNT-COF contents; **f** Comparison of mechanical properties and density of composite films with other reported films; **g** Gaussian calculations results based on PI-COFs + PAA; **h** Gaussian calculation results for MWCNT-COF + PAA at the middle of the MWCNT; **i** Gaussian calculation results for MWCNT-COF + PAA at the endpoint of the MWCNT

The stress–strain properties of PI films, COFs/PI films, and MWCNT-COF/PI composite films are presented in Figs. S7a, b and 5a–f. The mechanical properties of the composite films show significant improvement with the incorporation of MWCNT-COF. For example, the 0.5 wt% MWCNT-COF/PI composite film (Fig. 5d) demonstrates a tensile strength of 86.82 MPa, reflecting a 20.6% increase in comparison with the bare PI film, which exhibits a tensile strength of 72.01 MPa. Further enhancements are observed with increased MWCNT-COF content. The tensile strength of the 1 and 2 wt% MWCNT-COF/PI composite films increases by 26.59% to 91.16 MPa, and by 35.44% to 97.53 MPa, respectively. The functionalization of MWCNTs has proven to be an effective strategy for mitigating agglomeration and enhancing load transfer at the MWCNT-matrix interface. These modified MWCNTs act as both rigid crosslinkers and linear reinforcements, effectively minimizing the shrinkage of PI during the preparation process. Moreover, the MWCNT-COF coaxial hybrid nanomaterials are organized in a neat, porous structure, evenly distributed throughout the PI matrix. The adjacent MWCNTs form a 3D interconnected network, further enhancing the mechanical properties of the films. These results reveal that the in situ covalent growth of di-imide COF materials on the outer wall of MWCNTs effectively addresses challenges such as poor interface bonding, uneven dispersion, and structure instability commonly encountered in traditional physically mixed composites. Consequently, the ultra-thin composite films exhibit improved mechanical matching strength. The Young’s modulus results for bare PI and MWCNT-COF/PI composite films (Fig. 5e) display a clear enhancement trend, with values significantly increasing as the MWCNT-COF hybrid content rises. It has been established that the relationship between the Young’s modulus (GPa) and the volume fraction (%) of MWCNTs can be accurately described by polynomial equations [26]. Based on the following Eq.(1), the polymer Young’s modulus for MWCNTs-filled films is a function of the MWCNT volume fraction (${V}_{CNT}$) [26]:

1 $$E = 1.09464 + 0.67723V_{{{\mathrm{CNT}}}} - 0.28276V_{{{\mathrm{CNT}}}}^{2}$$

where ${V}_{CNT}$ represents the volume fraction (%) of MWCNTs. According to Ref. [26], the percentage increase in Young’s modulus is estimated to reach 100% at a CNT volume fraction of approximately 2%. The MWCNT volume fractions investigated in our study fall within this estimated range, thereby validating the applicability of Eq. (1) to our MWCNT-COF/PI composite system.

Although significant theoretical work has been conducted to predict the mechanical properties of MWCNT-reinforced composites, some of these models are quite complex, particularly for nanocomposites [15–17]. A modified form of mixing rules and Halpin–Tsai equations has been specifically adapted to predict the Young’s modulus of MWCNT-COF/PI composites [26]. These models take into account both the volume fraction of MWCNTs and the length-to-diameter ratio to estimate the Young’s modulus of the composite. According to the mixing law, it is assumed that MWCNT-COF and PI are thoroughly integrated, with MWCNT-COF evenly distributed within the PI matrix, resulting in a uniform stress distribution throughout the composite films. In the case of MWCNT-COF/PI composites, it is assumed that the MWCNT-COF adopts a randomly oriented, short fibrous packing arrangement [26, 27].

Based on the modified form of model [26], the composite modulus is expressed as:

2 $$\begin{array}{*{20}c} {{\mathrm{E}}_{{\mathrm{C}}} { = }\left( {{\upeta}_{{1}} {\upeta}_{{0}} {\mathrm{E}}_{{{\mathrm{CNT}}}} {\text{ - E}}_{{{\mathrm{PI}}}} } \right){\mathrm{V}}_{{{\mathrm{CNT}}}} {\text{ + E}}_{{{\mathrm{PI}}}} { }} \\ \end{array}$$

where ${\eta}_{1}$ is length efficiency factor, ${\eta}_{1}=1-\frac{{\mathrm{Tanh}}(a\bullet (l/d))}{(a\bullet (l/d))}$, $a=\sqrt{\frac{-3{E}_{PI}}{2{E}_{CNT}{\mathrm{ln}}{(V}_{CNT})}}$ and ${\eta}_{0}$ is orientation efficiency factor, ${\eta}_{0}=0.2$, for randomly oriented fillers.

The equations above are derived for randomly oriented composite materials. The primary reason the modulus increases linearly with the volume fraction is that ${V}_{C}$ correspondingly increases with the addition of MWCNT-COF in the PI matrix (Fig. 5d), indicating that the assumptions made to derive the equations align closely with experimental conditions. Moreover, the tensile load is effectively transferred to the nanotubes through the MWCNT-COF/PI interface. As COFs grow on the surface of the MWCNTs, the tube diameter (${\eta}_{1}$) increases, which in turn causes ${E}_{C}$ value to rise with the increase in both ${V}_{C}$ and ${\eta}_{1}$.

From Fig. 5f and Table 1, the mechanical and density properties of the MWCNT-COF/PI films developed in this work surpass those of previously reported PI-based films [28–33], further highlighting their promising application prospects in various high-performance fields. In addition, Gaussian computational studies conducted at the same theoretical level provide a comprehensive analysis of the PI-COFs + PAA and MWCNT-COF + PAA systems. The results indicate that the incorporation of COF-grafted MWCNTs significantly enhances material stability, as shown in Fig. 5g–i. A comparison of the relative energies per unit model reveals that the structure formed by the reaction of PI-COFs at the middle of MWCNT exhibits lower energy (approximately 0.552715 Hartree lower than that of the endpoint structure), making it thermodynamically more stable. However, transition state calculations demonstrate that the endpoint site, due to its structural defects and high curvature, possesses a lower activation energy barrier. This suggests that the reaction is kinetically controlled and preferentially occurs at the endpoint sites [34]. Furthermore, electronic structure analysis shows that the introduction of MWCNTs effectively extends the conjugated system, resulting in a reduced HOMO–LUMO gap. This is consistent with the theoretical expectation that enhanced conjugation leads to greater electron delocalization [35]. Collectively, these findings demonstrate that COF-grafted MWCNTs effectively modify the PAA composite material by synergistically enhancing its thermodynamic stability and kinetic reactivity while optimizing its electronic structure.

**Table 1:** Mechanical and density properties of PI substrates compared with previous reports

Sample	Stress (MPa)	Young’s modulus (GPa)	Density (g cm^–3^)	References
Fluorine-based dianhydrides PI	82	2.0	1.51	[28]
Bare PI	160	4.1	1.43	[29]
Bare PI	90	1.1	1.2	[30]
hydroxyl and bis-tert-butyl PI	74	2.0	1.29	[31]
BPDA-BAPP PI	95	1.0	1.30	[32]
Cu-PI	132	2.33	1.23	[33]
**1 wt% MWCNT-COF/PI**	**90.21**	**1.41**	**0.81**	**This Work**
**2 wt% MWCNT-COF/PI**	**97.53**	**1.53**	**0.73**	**This Work**

Bold is used to highlight the performance metrics of this work (our method), serving as a visual emphasis to distinguish our results from those of the other compared approaches

Moreover, the cross sectional SEM images of MWCNT-COF/PI composite films after tensile fracture were further examined, as shown in Fig. S8a–f. It can be observed that the addition of the MWCNT-COF hybrid facilitates load transfer by creating additional mechanical interlocking sites. During the film stretching, the strong adhesive interactions between MWCNT-COF and the polymer matrix generate stress. Compared to bare PI films, MWCNT-COFs predominantly fracture near the surface of the PI matrix, leading to local plastic deformation and a rough fracture surface. This behavior indicates an improved interfacial adhesion between MWCNT-COF and the PI matrix, making it more challenging for MWCNT-COFs to be pulled out during film facture. These results demonstrate that the MWCNT-COF hybrid material is evenly dispersed throughout the films, promoting more uniform force transfer during stretching [36]. As is well known, the high surface-to-volume ratio of nanofillers significantly enhances material properties [37]. The increased surface-to-volume ratio of MWCNT further augments the filler–matrix interaction. Additionally, the uniform distribution of the nanofillers improves load transfer during the tensile process. While COFs introduce ordered pores into the films, their presence on the surface of MWCNT further enhances mechanical performance. COFs act as covalent bridges between MWCNT and the PI matrix, improving interfacial bonding and facilitating load transfer. The formation of covalent bonds between the COFs and the PI matrix enhances mechanical properties by improving load transfer between MWCNTs and the surrounding matrix. Moreover, the larger surface area of MWCNT-COFs per unit volume enables more efficient load transfer to the nanotube network, greatly enhancing the mechanical properties of the composite films.

### Photoelectric Conversion Properties of Flexible Solar Cells

**Fig. 6:**
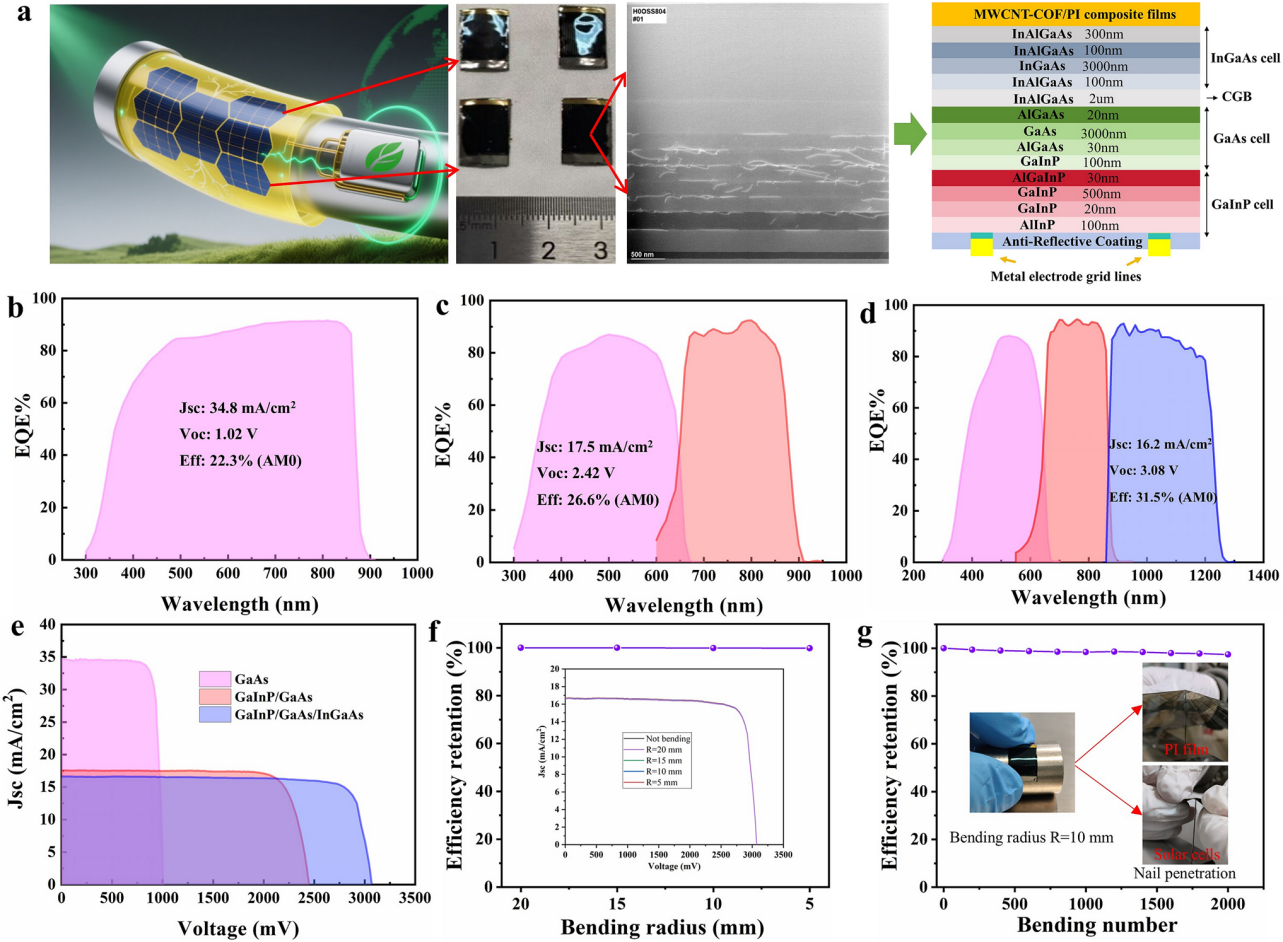
Optical and *J–V* characteristics of ultra-thin GaAs solar cells. **a** GaAs solar cells fabricated via the inversion transfer process and their STEM cross sectional structure; EQE of **b** a single-junction GaAs solar cell, **c** a GaInP/GaAs dual-junction solar cell, **d** a GaInP/GaAs/InGaAs triple-junction solar cell; **e** *J–V* characteristics of GaAs solar cells with different junctions under standard test conditions (AM0, 25 °C)); Efficiency retention of flexible solar cells **f** under various bending radii and **g** repeated bending cycles at a bending radius of R = 10 mm. The inset in **f** is the *J–V* curves of solar cells obtained at different bending states, and in **g** is the photograph of bending radius at R = 10 mm

To demonstrate the practical application of ultra-thin PI films modified with MWCNT-COF, GaAs solar cells were fabricated using a 6 µm-thick PI film substrate, employing epitaxial inverted growth and low-temperature metal bonding technology. Figure 6a presents the physical image and cross sectional view of the flexible GaAs solar cell, which was fabricated using the MWCNT-COF/PI composite films through a flip-chip transfer process [38]. It can be observed that the flexible solar cell exhibits minimal wrinkling, indicating that the MWCNT-COF-modified PI substrate provides high toughness. This enhanced flexibility enables the MWCNT-COF/PI composite film to effectively support the epitaxial layer of the solar cell, leading to improved photoelectric performance. Figure 6b-e illustrates the current–voltage (*J-V*) characteristics and external quantum efficiency (EQE) of the GaAs solar cell, measured using standard solar cell testing equipment under standard test conditions (AM0, 25 °C) [39]. The EQE of single-junction, double-junction, and triple-junction GaAs solar cells based on the MWCNT-COF/PI composite film substrate in the 300–1200 nm wavelength range all exceed 90%, demonstrating remarkable absorption across this spectrum. Furthermore, the triple-junction GaAs solar cell achieves the highest current density (*J*_sc_ = 16.2 mA cm^−2^), an open-circuit voltage of 3.08 V, and a fill factor of 0.85. As shown in Table S3, when compared to other solar cells with different substrates [4, 40–44], the ultra-thin dual-junction and triple-junction GaAs solar cells based on MWCNT-COF/PI with conversion efficiency of 26.6% and 31.5% demonstrates exceptional specific powers of 8998 and 7749 W kg^−1^, respectively.

Notably, as shown in Fig. 6f, the ultra-thin flexible GaAs solar cell exhibits almost unchanged *J–V* curves, corresponding to high efficiency retention at different bending radii. More importantly, when the flexible solar cells were subjected to continuous bending for 2000 cycles at a bending radius of R = 10 mm, they still demonstrated excellent efficiency retention (Fig. 6g), highlighting their significant potential for flexible and lightweight photovoltaic systems in both terrestrial and space applications. As illustrated, this ultra-thin and robust PI film structure can withstand lateral tensile forces and longitudinal nail penetration to a certain extent, owing to the anchoring of robust MWCNTs and covalently bonded COF layers within the PI substrate.

## Conclusions

In summary, ultra-thin, lightweight, and robust PI films were successfully prepared through the in situ incorporation of MWCNT-COF coaxial hybrid materials for ultra-thin, high-specific-power GaAs solar cells. The synergy between MWCNTs and a covalently anchored porous COF layer, combined with the thermally stable molecular structure of PI, conferred remarkable strength and reduced weight to the films. The resulting ultra-thin porous MWCNT-COF/PI composite films, with a thickness of 6 µm, exhibited a high Young’s modulus of 1.53 GPa and a tensile strength of 97.53 MPa, along with a low density of 0.73 g cm^−3^. Compared to bare PI films, these composites demonstrated a 109% increase in Young’s modulus and a 35.4% enhancement in tensile strength, attributed to the anchoring effects of MWCNTs and the covalently bonded COF layers, while also achieving a 45.1% reduction in density. This underscores the critical role of the 3D network formed by MWCNT-COF coaxial hybrids in enhancing the performance of PI films. Additionally, Gaussian computational simulations confirmed that COF-grafted MWCNTs effectively modified the PAA composite material, further improving the mechanical properties of PI. Flexible dual-junction and triple-junction GaAs solar cells, utilizing the 6-µm-thick MWCNT-COF/PI composite films as substrates, achieved a photoelectric conversion efficiency of 26.6% and 31.5% with high specific powers of 8998 and 7749 W kg^−1^, respectively. The design concept presented in this work may pave the way for the development of ultra-thin, lightweight, and robust films applicable in various fields.

## Supplementary Information

Below is the link to the electronic supplementary material.

Supplementary file1 (DOCX 3179 kb)
