# Supplementary material for: Covalent Organic Framework-Anchored Carbon Nanotubes Enabling Ultra-Thin Robust Polyimide Films for High-Specific-Power Flexible GaAs Solar Cells
Source: Nanomicro Lett. 2026 Aug 10;19:31. doi: 10.1007/s40820-026-02326-1 (PMC13457540; doi:10.1007/s40820-026-02326-1)
Supplement: Supplementary file 1 [file 40820_2026_2326_MOESM1_ESM.docx]

Supporting Information for

**Covalent Organic Framework-Anchored Carbon Nanotubes Enabling** **Ultra-Thin Robust Polyimide Films for High-Specific-Power Flexible GaAs Solar Cells**

Bin Li^1^, Zhejun Guo^2^, Qin Wang^3^, Maoshu Yin^2^, Xianglei Shi^2^, Lu Yao^5^, Chongjing Lu^4^, Yanpeng Zhang^1^, Wenwei Xu^2^, Liying Jiang^1^, Lijie Sun^2, *^, Feng Shao^5, *^, Xuan Liu^3, *^ and Nantao Hu^4, *^

^1^ Henan Key Laboratory of Information Functional Materials and Sensing Technology, Zhengzhou University of Light Industry, Zhengzhou 450002, P. R. China

^2^ Research Center for Photovoltaics, Shanghai Institute of Space Power-Sources, Shanghai 200245, P. R. China

^3^ College of Engineering Science and Technology, Shanghai Ocean University, Shanghai 201306, P. R. China

^4^ Key Laboratory of Intelligent Creation for Extreme Energy Materials (Ministry of Education), Shanghai Jiao Tong University, Shanghai 200240, P. R. China

^5^ Faculty of Chemical Engineering and Energy Technology, Shanghai Institute of Technology, Shanghai 201418, P. R. China

*Corresponding authors. E-mail: [sunlijielu@163.com](mailto:sunlijielu@163.com) (Lijie Sun); [shaofeng@sit.edu.cn](mailto:shaofeng@sit.edu.cn) (Feng Shao); [xliu@shou.edu.cn](mailto:xliu@shou.edu.cn) (Xuan Liu); [hunantao@sjtu.edu.cn](mailto:hunantao@sjtu.edu.cn) (Nantao Hu)

**S1 Supplementary Text**

***Calculation of porosity***

The quantitative porosity analysis can use the methods:

(i) Density-based calculation [S1-S3]:

The porosity was calculated using the equation:

$\text{Porosity}=(1-\rho_{\text{composite}}/\rho_{\text{theoretical}})\times100\%$	(S1)

where $\rho_{\text{theoretical}}$ was determined using the rule of mixtures based on the mass fractions and true densities of the composite film ($\rho_{\text{composite}}=0.73{g/cm}^{3}$), PI ($\rho=1.35\text{ }\text{g/cm}^{3}$), MWCNTs ($\rho=2.0\text{ }\text{g/cm}^{3}$), and COF ($\rho=1.30\text{ }\text{g/cm}^{3}$). The calculation yields an approximate porosity of 46.7% for the 2 wt.% MWCNT-COF/PI composite film. Detailed calculations and results for other mixing ratios are provided in Table S1. This quantitative result confirms that the observed density reduction (from 1.31 g/cm³ to 0.73 g/cm³) is attributable to the highly porous structure introduced by the MWCNT-COF network.

(ii) SEM image analysis:

We further analyzed cross-sectional SEM images using ImageJ software to statistically determine the pore area fraction based on the Fig. S6(a-f). The results show a porosity of 41.1% for the 2 wt.% MWCNT-COF at different mixing ratios, which is consistent with the density-based calculation. This SEM image analysis method was employed to evaluate the cross-sectional SEM images and to compare the corresponding pore structures of different mixing ratios of MWCNT-COF. Additionally, the results for other mixing ratios and detailed calculations can be found in Table S2.

**S2 Supporting Figures and Tables**

**Fig. S1** Schematic illustration of the fabrication process for MWCNT-COF and MWCNT-COF/PI composite films: **a** Synthesis of MWCNT-COF, **b** Fabricating of ultrathin porous MWCNT-COF/PI composite films

**Fig. S2** SEM images of bare **a** PI and MWCNT-COF/PI composite films with **b** 0.1 Wt.%, **c** 0.2 Wt.%, **d** 0.5 Wt.%, **e** 1 Wt.% and **f** 2 Wt.% MWCNT-COF

**Fig. S3** Contact angles of bare PI and MWCNT-COF/PI composite films

**Fig. S4** SEM images of the cross-section morphology of a bare PI and COFs/PI films: **a** Bare PI, **b** 1 Wt.% COFs/PI, **c** 2 Wt.% COFs/PI, **d** 3 Wt.% COFs/PI, **e** 4 Wt.% COFs/PI and **f** 5 Wt.% COFs/PI

**Fig. S5** Characterization results of bare PI and MWCNT-COF/PI composite films. SEM images of the cross-section morphology of **a** bare PI and composite films with different proportions of MWCNT-COF: **b** 0.1 Wt.%, **c** 0.2 Wt.%, **d** 0.5 Wt.%, **e** 1 Wt.% and **f** 2 Wt.%

**Fig. S6** Comparison diagrams of cross-sectional SEM images and corresponding pore structures based on SEM image analysis for different mixing ratios of MWCNT-COF: **a** 0.1 Wt.%, **b** 0.2 Wt.%, **c** 0.5 Wt.%, **d** 1 Wt.%, **e** 2 Wt.%

**Fig. S7 a** Stress-Strain curves of PI films and **b** Young's modulus of PI films

**Fig. S8** SEM images of a bare PI and MWCNT-COF/PI composite films with different proportions: **b** 0.1 Wt.%, **c** 0.2 Wt.%, **d** 0.5 Wt.%, **e** 1 Wt.%, **f** 2 Wt.% after tensile fracture

**Table S1** The porosity of PI substrates with different mixing ratios of MWCNT-COF

| Sample | Density of film (g/cm^3^) | Porosity |
| --- | --- | --- |
| 0.1wt.% MWCNT-COF/PI | 1.31 | 4.4% |
| 0.2 wt.% MWCNT-COF/PI | 1.18 | 13.9% |
| 0.5 wt.% MWCNT-COF/PI | 0.91 | 33.6% |
| 1 wt.% MWCNT-COF/PI | 0.81 | 40.9% |
| 2 wt.% MWCNT-COF/PI | 0.73 | 46.7% |

**Table S2** The porosity of PI substrates with different mixing ratios of CNTs by SEM image analysis

| Sample | Density of film (g/cm^3^) | Pore area  (nm^2^) | Total area  (nm^2^) | Porosity |
| --- | --- | --- | --- | --- |
| 0.1wt.% MWCNT-COF/PI | 1.31 | 487596.7 | 3549.9×2699.9 | 5.1% |
| 0.2 wt.% MWCNT-COF/PI | 1.18 | 475358.0 | 2273.3×1540.0 | 13.5% |
| 0.5 wt.% MWCNT-COF/PI | 0.91 | 854646.9 | 2164.4×1277.78 | 30.9% |
| 1 wt.% MWCNT-COF/PI | 0.81 | 1246823.9 | 2258.3×1523.2 | 36.2% |
| 2 wt.% MWCNT-COF/PI | 0.73 | 1420966.2 | 2264.9×1525.5 | 41.1% |

**Table S3** Parameters of GaAs solar cells with different types of substrates and MWCNT-COF/PI composite films

| Sample | Photoelectric conversion efficiency (AM0) | Areal density (g/m^2^) | Specific-power (W/kg) | References |
| --- | --- | --- | --- | --- |
| Triple-junction GaAs/Si solar cell (Silicon) | 30.3% | 200-500 | 820-2050 | [S4] |
| Triple-junction perovskite/perovskite/silicon solar cell (Silicon) | 26.1% | 3000-3300 | 107-118 | [S5] |
| Triple-junction GaAs solar cell (Bare Cu) | 33.47% | 230 | 1968 | [S6] |
| Four-junction GaAs solar cell (Bare PI) | 23.4% | 467 | 678 | [S7] |
| Dual-junction GaAs solar cell (Bare PI) | 24.9% | 50 | 6736 | [S8] |
| Triple-junction GaAs solar cell (Bare PI) | 30.2% | 132 | 3093 | [S9] |
| Dual-junction GaAs solar cell (**MWCNT-COF/PI**) | **26.6%** | **40** | **8998** | **This Work** |
| Triple-junction GaAs solar cell (**MWCNT-COF/PI**) | **31.5%** | **55** | **7749** | **This Work** |

**Supplementary References**

[S1] M.A. Silva, M.N.N. Miranda, Estimation of properties of ternary mixtures of solids using the mixing rule. Powder Technol. **134**(1–2), 16–23 (2003). <https://doi.org/10.1016/S0032-5910(03)00131-1>
[S2] H. Akbar, et al. Density and porosity calculation of composites refer to the rule of mixture (ROM). Procedia Struct. Integr., **27**, 62-68 (2020). <https://doi.org/10.1016/j.prostr.2020.07.009>
[S3] M. Ravikumar, R. Nail, Impact of nano sized SiC and Gr on mechanical properties of aerospace grade Al7075 composites. Frat. Ed Integrità Strutturale **16**(62), 439–447 (2022). <https://doi.org/10.3221/igf-esis.62.30>
[S4] R. Cariou, J. Benick, F. Feldmann, O. Höhn, H. Hauser et al., III–V-on-silicon solar cells reaching 33% photoconversion efficiency in two-terminal configuration. Nat. Energy **3**(4), 326–333 (2018). <https://doi.org/10.1038/s41560-018-0125-0>
[S5] F. Xu, E. Aydin, I. Yavuz, C. Deger, E. Ugur et al., Stabilized perovskite phases enabling efficient perovskite/perovskite/silicon triple-junction solar cells. Nat. Mater. **25**(2), 259–266 (2026). <https://doi.org/10.1038/s41563-025-02367-8>
[S6] M. Yu, J. Long, Q. Sun, Z. Chen, X. Wu et al., Achieving flexible higher efficiency GaInP/GaAs/InGaAs solar cells by 40-period quantum well superlattices. Nano Energy **136**, 110718 (2025). <https://doi.org/10.1016/j.nanoen.2025.110718>
[S7] X. Huang, J. Long, D. Wu, S. Ye, X. Li et al., Flexible four-junction inverted metamorphic AlGaInP/AlGaAs/In_0.17_Ga_0.83_As/In_0.47_Ga_0.53_As solar cell. Sol. Energy Mater. Sol. Cells **208**, 110398 (2020). <https://doi.org/10.1016/j.solmat.2020.110398>
[S8] T.S. Kim, H.J. Kim, D.-M. Geum, J.-H. Han, I.S. Kim et al., Ultra-lightweight, flexible InGaP/GaAs tandem solar cells with a dual-function encapsulation layer. ACS Appl. Mater. Interfaces **13**(11), 13248–13253 (2021). <https://doi.org/10.1021/acsami.1c00006>
[S9] J. Schön, G.M.M.W. Bissels, P. Mulder, R.H. van Leest, N. Gruginskie et al., Improvements in ultra-light and flexible epitaxial lift-off GaInP/GaAs/GaInAs solar cells for space applications. Prog. Photovolt. Res. Appl. **30**(8), 1003–1011 (2022). <https://doi.org/10.1002/pip.3542>
